# Pre-Clinical Characterization of Dacomitinib (PF-00299804), an Irreversible Pan-ErbB Inhibitor, Combined with Ionizing Radiation for Head and Neck Squamous Cell Carcinoma

**DOI:** 10.1371/journal.pone.0098557

**Published:** 2014-05-22

**Authors:** Justin P. Williams, Inki Kim, Emma Ito, Wei Shi, Shijun Yue, Lillian L. Siu, John Waldron, Brian O'Sullivan, Kenneth W. Yip, Fei-Fei Liu

**Affiliations:** 1 Ontario Cancer Institute, University Health Network, Toronto, Ontario, Canada; 2 Asan Institute for Life Science, Asan Medical Center, Seoul, Republic of Korea; 3 Division of Medical Oncology, Princess Margaret Cancer Centre and University of Toronto, Toronto, Ontario, Canada; 4 Department of Medical Biophysics, University of Toronto, Toronto, Ontario, Canada; 5 Department of Radiation Oncology, Princess Margaret Cancer Centre and University of Toronto, Toronto, Ontario, Canada; Ludwig-Maximilians University, Germany

## Abstract

Epidermal growth factor receptor (EGFR) is over-expressed in nearly all cases of squamous cell carcinoma of the head and neck (SCCHN), and is an important driver of disease progression. EGFR targeted therapies have demonstrated clinical benefit for SCCHN treatment. In this report, we investigated the pre-clinical efficacy of Dacomitinib (PF-00299804), an irreversible pan-ErbB inhibitor, both alone and in combination with ionizing radiation (IR), a primary curative modality for SCCHN. One normal oral epithelial (NOE) and three SCCHN (FaDu, UT-SCC-8, UT-SCC-42a) cell lines were used to conduct cell viability, clonogenic survival, cell cycle, and immunoblotting assays *in vitro*, using increasing doses of Dacomitinib (10–500 nM), both with and without IR (2–4 Gy). The FaDu xenograft model was utilized for tumor growth delay assays *in vivo*, and immunohistochemical analyses were conducted on extracted tumors. A dose-dependent reduction in cell viability and clonogenic survival after Dacomitinib treatment was observed in all three SCCHN models. Treatment led to a significant reduction in EGFR signalling, with a subsequent decrease in phosphorylation of downstream targets such as ERK, AKT, and mTOR. *In vivo*, Dacomitinib treatment delayed tumor growth, while decreasing phospho-EGFR and Ki-67 immunoexpression. These effects were further enhanced when combined with IR, both *in vitro* and *in vivo*. The preclinical data support the further evaluations of Dacomitinib combined with IR for the future management of patients with SCCHN.

## Introduction

Squamous cell carcinoma of the head and neck (SCCHN) is the 6^th^ most common cancer world-wide, with approximately 600,000 new cases presenting each year [1]. Amongst these cases, approximately 60% of patients will present with locally advanced disease [1]. The standard management for these patients involves curative surgery or radiation therapy (RT), possibly combined with chemotherapy [2]. Despite such multi-modal approaches, the 5-year overall survival for patients with locally advanced SCCHN (LA-SCCHN) has remained stable, and modest, at around 40–50% [1]. Furthermore, patients presenting with LA-SCCHN are more than twice as likely to experience loco-regional failure as opposed to distant metastasis; the 5 year loco-regional recurrence and distant metastasis rates are 50–60%, and 20–30%, respectively [3]. Taken together, this reinforces the need to develop improved therapeutic strategies.

The epidermal growth factor receptor (EGFR, HER-1/ErbB1) is one of four closely related receptor tyrosine kinases within the ErbB family. Activation of this receptor is initiated by ligand binding, followed by homo- or hetero-dimerization with other ErbB members, with subsequent phosphorylation of intracellular tyrosine kinase domains. EGFR activation leads to downstream signalling *via* the PI3K/AKT and the RAS/RAF/MAPK pathways. These signalling cascades are responsible for initiating a number of cellular processes associated with SCCHN disease progression, including tumor growth, invasion, angiogenesis, and metastasis [4], [5]. EGFR is over-expressed in a variety of human malignancies, including lung, gastric, colorectal, and breast cancers, as well as SCCHN [6]–[9]. In SCCHN, EGFR is over-expressed in up to 90% of cases, associated with poor prognosis, resistance to RT/chemotherapy, and reduced overall survival [10], [11].

Currently, there exists two major classes of EGFR targeted anti-cancer agents: (a) tyrosine kinase inhibitors (TKIs), such as Gefitinib and Erlotinib, which target the intracellular adenosine triphosphate (ATP) binding sites of the receptor; and (b) monoclonal antibodies against EGFR, most notably Cetuximab and Panitumumab, which target the extracellular ligand binding site of the receptor. There have been many pre-clinical studies documenting the role of EGFR targeting for SCCHN management [12], [13], culminating in the positive Phase III trial in support of Cetuximab plus RT [14], which has transformed clinical management. However, *de novo* or acquired resistance to this strategy has become an important clinical issue, emphasising the need to explore alternate therapeutic strategies [15].

Dacomitinib (PF-00299804) is an orally available, irreversible, pan-ErbB TK inhibitor that targets the ATP binding site located on the intracellular domain of the EGFR, ErbB2, and ErbB4 receptors [16]. The efficacy of Dacomitinib has been previously evaluated in gastric and non-small cell lung cancer (NSCLC) models, wherein the compound inhibited tumor cell proliferation and delayed tumor growth *in vivo* [17], [18]. More recently, the anti-proliferative effects of Dacomitinib have been demonstrated in a panel of SCCHN cell lines [19]. Phase I, II, and III clinical trials with Dacomitinib have been completed or are currently underway for a variety of cancer types, including one current (NCT01449201), and one recently completed (NCT00768664), multi-centre, phase II clinical trials for patients with SCCHN [20]. Completed trials have revealed that Dacomitinib treatment is well tolerated in patients, and partial responses have been observed in gefitinib/erlotinib refractory NSCLC patients [21], [22].

To date however, the effects of Dacomitinib combined with ionizing radiation (IR) have yet to be established in SCCHN. Based on the importance of EGFR in SCCHN progression, we hypothesized that Dacomitinib would effectively reduce tumor viability and enhance IR cytotoxicity in SCCHN models. Herein, we present the first evaluation of Dacomitinib with IR in both *in vitro* and *in vivo* SCCHN models.

## Materials and Methods

### Ethics Statement

All animal experiments were conducted in accordance to guidelines of the Animal Care Committee (ACC) at the University Health Network (Toronto, Canada). The protocol was approved by the Animal Care Committee (ACC) at the University Health Network (Protocol Number: 342.22). Injections were performed under Isoflurane anesthetic and all efforts were made to minimize suffering. Mice were sacrificed under general anesthetic (isoflurane, as above) using carbon dioxide and then cervical dislocation, as advised by the ACC.

### Cell Lines

Three human SCCHN cell lines were utilized: FaDu (hypopharyngeal squamous carcinoma; American Type Culture Collection), UT-SCC-8 and UT-SCC-42a (laryngeal squamous cell carcinoma); the latter two lines were a generous gift from R. Grenman (Department of Otorhinolaryntology-Head and Neck Surgery, University of Turku, Finland) [23]. FaDu cells were maintained in Minimum Essential Medium, supplemented with 10% Fetal Bovine Serum (FBS), 1.5 g/L bicarbonate, and 1 mM pyruvate. UT-SCC cells were maintained in Dulbecco's Modified Eagle's Medium, supplemented with 10% FBS. The normal oral epithelial (NOE) cell line was maintained in normal human oral epithelial media (Celprogen). All the cell lines were maintained at 37°C, 5% CO_2_; authenticated using the AmpFISTR Identifiler PCR amplification kit (Life Technologies), and routinely tested for *mycoplasma* (Mycoalert detection kit; Lonza Group Ltd).

### Compound Dilutions

Dacomitinib was provided by Pfizer Canada, Inc. For *in vitro* studies, stock solutions of Dacomitinib were diluted in 100% Dimethyl Sulfoxide (DMSO) at a concentration of 10 mM, and were stored at −80°C. Subsequent working solutions (0.01–2.0 µM) were prepared in media. As a negative control (untreated), DMSO was added to media to a concentration of 0.01%, which corresponded to the DMSO concentration found in the highest Dacomitinib treatment group. For *in vivo* studies, stock solutions of Dacomitinib (1 mg/mL) were prepared in 100% DMSO and stored at −80°C.

### RNA Extraction and Quantitative Real-Time PCR

Cells were seeded in 6-well plates at a density of 3×10^5^ cells per well. Forty-eight hours post-seeding, cells were lysed for total RNA extraction using the RNeasy Mini Kit (Qiagen). Reverse transcription was performed using SuperScript III reverse transcriptase (Life Technologies) according to the manufacturer's specifications. Quantitative Real-Time PCR (qRT-PCR) was performed using SYBR Green (Life Technologies) and a Perkin-Elmer/ABI Prism 7900 sequence detection system (PE Biosystems). Gene specific primers for EGFR, ErbB2, ErbB3, ErbB4, and β-actin were designed using Primer3 (NCBI; Table S1 in File S1). The mean fold change in mRNA expression was calculated using the 2^−ΔΔCt^ method [24].

### Radiation Treatments

For *in vitro* experiments, cells were irradiated at room temperature using a ^137^Cs unit (Gammacell 40 Extractor; Nordion International) at a dose rate of 0.84 Gy/min. Irradiation was administered 24 hours post-seeding, and within 1 hour following any drug treatment, unless stated otherwise. For *in vivo* experiments, mice were immobilized in a Lucite box, and the tumor-bearing leg was exposed to 225 kVp (13 mA) at a dose rate of 3.37 Gy/min using an X-ray irradiator C (X-RAD 225; Precision X-ray).

### Cell Viability Assay

The soluble tetrazolium salt [3-(4,5-dimethylthiazol-2-yl)-5-(3-carboxymethonyphenol)-2-(4-sulfophenyl)-2H-tetrazolium, inner salt (MTS); Promega Corp.] cell proliferation assay was used to assess Dacomitinib cytotoxicity, with or without IR. Cells were seeded in 96-well plates at a density of 2500 cells/well in 50 µL of media, and incubated for 24 hours. Working solutions of Dacomitinib or DMSO were prepared such that an additional 50 µL of media plus drug (or DMSO) was added to the corresponding wells. The MTS assay was performed 72 hours post-treatment, according to the manufacturer's specifications.

### Colony Formation Assay

Cells were seeded in 12-well plates, at a density of 100–1000 cells/well in 500 µL of media, and incubated for 24 hours. Working solutions of Dacomitinib or DMSO were prepared such that an additional 500 µL of media plus drug (or DMSO) was added to corresponding wells. Cells were incubated for an additional 10–16 days (Dacomitinib/media not refreshed), then fixed with 70% ethanol and stained with 0.1% methylene blue. The experimental endpoint was defined as the day in which greater than 75% of the colonies in the DMSO control wells contained greater than 50 cells per colony. Surviving fractions were calculated as the ratio of the number of colonies (defined as containing greater than 50 cells) in the treated wells to the number of colonies in the vehicle control wells.

### Western Blot Analysis

Cells were seeded at a density of 5×10^5^ cells/well in 6-well plates. Twenty-four hours post seeding, media was removed, cells were rinsed with PBS, and serum-free media containing Dacomitinib or DMSO was added to each well. Twenty-four hours post treatment, the EGFR ligand epidermal growth factor (EGF; Sigma), was added to one set of the treatment plates to a final concentration 20 ng/mL, then incubated for an additional 30 minutes. After EGF treatment, media from all plates was removed; cells were washed with PBS, and lysed at 4°C using a lysis buffer containing protease and phosphatase inhibitors (Roche). Twenty micrograms of extracted protein for each sample was separated on a 4–20% gradient polyacrylamide Tris-glycine gel (Life Technologies; Foster City, California) and transferred to PVDF membranes (Millipore). Membranes were incubated overnight at 4°C with specific antibodies against EGFR (#4267), phospho-EGFR (#3777), ERK (#4695), phospho-ERK (#4370), AKT (#4691), phospho-AKT (#4060), mTOR (#2983), phospho-mTOR (#2971), STAT3 (#9139), and phospho-STAT3 (#9145; all from Cell Signalling, 1∶1000 dilution). Blots were then incubated with horseradish peroxidase conjugated to anti-rabbit or anti-mouse antibody (1∶5000; Biorad), then analyzed with Supersignal West Pico Chemiluminescent system (Thermo Scientific).

### Cell Cycle Analysis

Cells were seeded at a density of 3×10^5^ cells/well in 6-well plates. Twenty-four hours post-seeding, cells were subjected to 1 of 4 possible treatments: negative control (0.01% DMSO), IR only (2 or 4 Gy), Dacomitinib only (10 or 100 nM), or Dacomitinib + IR. At 48 and 72 hours post-treatment, cells were harvested and stained with propidium iodide, as previously described [25]. Cells were processed using the BD FACSCalibur system and analysed using FlowJo software (Tree Star).

### Animal Experiments

Six to eight week old male BALB/c severe combined immune deficient (SCID) mice were obtained from the Animal Research Colony at the Ontario Cancer Institute. Mice were housed in the pathogen free Animal Facility at Princess Margaret Cancer Centre in cages with 5 mice per cage during tumor generation, and subsequently reduced to 3 mice per cage once selected for treatment. Mice had free access to food and water. FaDu cells (2.5×10^5^ cells per 100 µL media) were injected into the left gastrocnemius muscle of mice and tumor generation was monitored as previously described [26]. Once the tumor plus leg diameter (TLD) reached approximately 8 mm, the mice were randomly assigned to one of the following treatment groups: (a) negative control (100% DMSO); (b) Dacomitinib only; (c) negative control plus IR; or (d) Dacomitinib plus IR. Dacomitinib or negative control was administered orally (approximately 100 µL) once daily, for 5 consecutive days, using a 27-gauge oral gavage needle. Local IR treatments were administered on days 2 and 5, immediately following drug or negative control administration. TLD and mouse body weight were measured at least three times per week. Animals were sacrificed once the TLD reached 14–15 mm, as per ACC guidelines. To overcome logistical issues with completing a single, larger experiment, 3 smaller, independent experiments were completed with 3 mice per treatment group (total n = 9 for each group).

### Immunohistochemistry

Mice were treated as described above and then sacrificed 24 hours after the final treatment. Tumors were removed, immediately fixed in 10% formalin-PBS for 24 hours, placed in 70% alcohol for 48 hours, then paraffin embedded, and sectioned (5 µm). Immunohistochemistry was conducted using a purified mouse anti-human Ki-67 antigen (1∶100 dilution; DakoCytomation), as previously described [27]. Phospho-EGFR immunohistochemistry was performed using microwave antigen retrieval in combination with the Level-2 Ultra Streptavidin System and anti-p-EGFR antibody (1∶100 dilution; #3777, Cell Signalling), as previously described [28]. Terminal deoxynucleotidyl transferase-mediated dUTP nick end labelling (TUNEL) staining was assessed using the *In Situ* Cell Death Detection Kit (Roche Diagnostics).

Immunoscoring was performed as previously reported [28]; wherein any tumor nuclei expression for Ki-67 was considered positive. Phosphorylated EGFR was scored using three sections from three independent tumors, and the relative staining intensity was then calculated [28].

### Statistical Analysis

All experiments have been performed at least three independent times, and the data are presented as the mean ± SEM. Statistical significance between two treatment groups was determined using the Student's *t* test, one-way ANOVA, or two-way ANOVA. Statistical analyses and graphs were prepared using Graphpad Prism 5 software (Graphpad Software Inc.).

## Results

### EGFR is over-expressed in SCCHN cell lines

**Figure 1:**
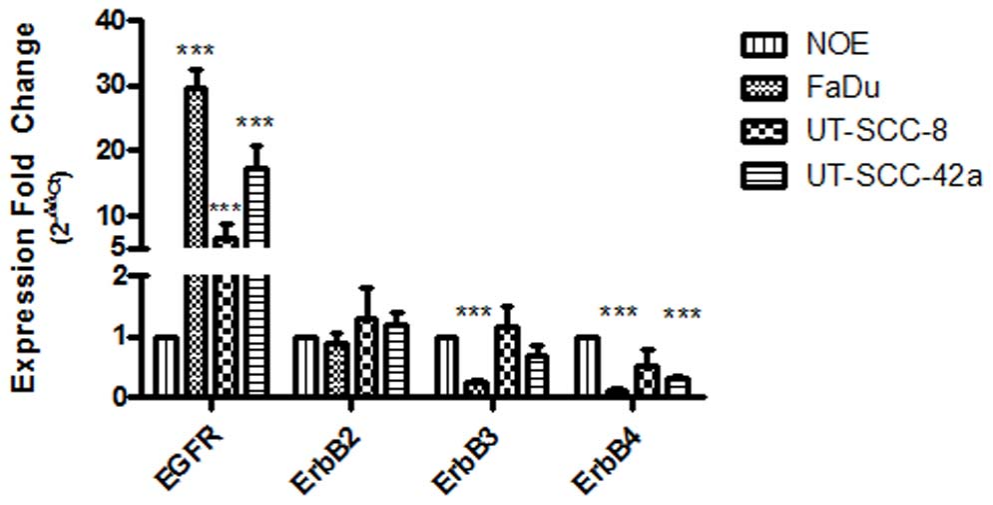
EGFR is over-expressed in SCCHN cell lines. qRT-PCR was performed to measure ErbB transcript levels in the three SCCHN cell lines, normalized to that of the normal oral epithelial (NOE) cells. Expression fold change was determined by the 2^−ΔΔCt^ method. ***p<0.001; student's *t* test.

The level of ErbB mRNA expression in SCCHN (FaDu, UT-SCC-8, UT-SCC-42a) *vs*. normal oral epithelial (NOE) cell lines was first determined using qRT-PCR. A significant over-expression of EGFR was observed in all three SCCHN cell lines (Figure 1), with the highest level demonstrated in the FaDu cells, exhibiting a 30-fold increase as compared to the NOE's. In contrast, ErbB2, ErbB3, or ErbB4 were not significantly over-expressed in any of these three SCCHN cell lines. As a result, subsequent experiments were focused primarily on the EGFR pathway and its downstream targets.

### Combining Dacomitinib and IR reduced SCCHN viability and colony formation in vitro

**Figure 2:**
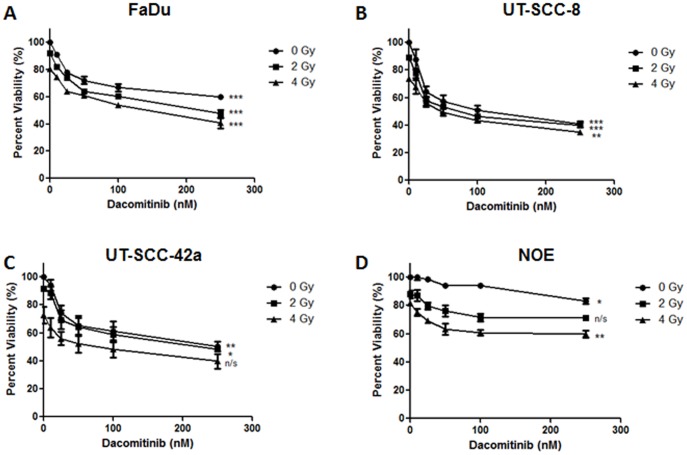
Dacomitinib demonstrated *in vitro* efficacy in EGFR over-expressing SCCHN cell lines. (A to D) Growth inhibition of three SCCHN and the NOE cell lines, treated with Dacomitinib, with or without IR. (B) FaDu; (C) UT-SCC-8; (D) UT-SCC-42a; and (E) NOE cells were treated with Dacomitinib (0, 10, 25, 50, 100, or 250 nM) alone, or in combination with IR (0, 2, or 4 Gy). MTS assays were conducted 72 hours post-treatment. The graphs represent data from 3 independent experiments, with the mean ± SEM reported. *p<0.05, **p<0.01, ***p<0.001; one-way ANOVA. No statistically significant difference between curves for Figure 2A-D; two-way ANOVA. Combination of Dacomitinib plus RT did not result in a synergistic interaction for any of the dosing regimens in all three SCCHN cell lines (Chou-Talalay Method).

The cytotoxicity of Dacomitinib (0–250 nM) was subsequently examined on the three SCCHN cell lines, as well as the NOE cells, using the MTS assay. As shown in Figure 2, 72 hours' treatment with Dacomitinib alone resulted in a dose-dependent reduction in cell viability across all three SCCHN cell lines. The UT-SCC-8 and UT-SCC-42a cells exhibited a greater sensitivity to Dacomitinib with similar IC25 (concentration of Dacomitinib that inhibits cell growth by 25%, as compared to the untreated control) values at 25 nM. The FaDu cells were less sensitive with an IC25 value of approximately 50 nM. The NOE's were much more resistant to Dacomitinib treatment with an IC25 of >250 nM. The combination of Dacomitinib (10–250 nM) with concurrent IR (2 or 4 Gy) resulted in a further reduction in cell viability for all three SCCHN cell lines. The greatest reductions were observed when 4 Gy was combined with 250 nM of Dacomitinib, resulting in a 50–65% reduction in viability across all three SCCHN cell lines.

**Figure 3:**
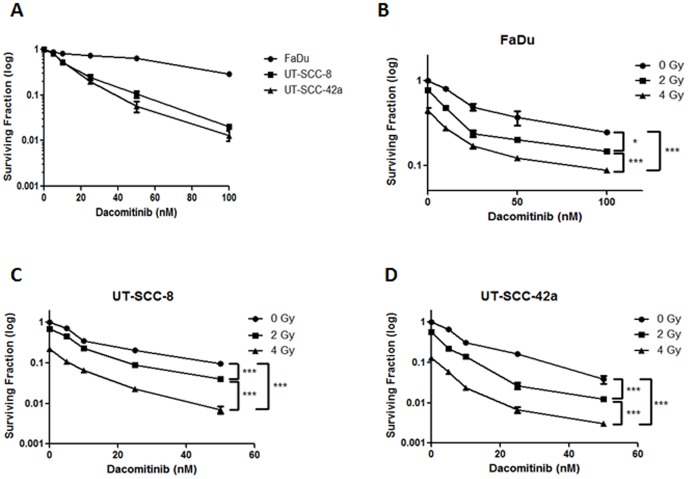
Dacomitinib plus ionizing radiation reduced clonogenic potential of SCCHN cell lines. (A) Relative sensitivities of SCCHN cell lines treated with Dacomitinib alone. p<0.001 for all points on each curve, as compared to negative control; Student's *t* test. (B–D) FaDu, UT-SCC-8, and UT-SCC-42a cells were treated with Dacomitinib (0–100 nM) in combination with IR (0, 2, or 4 Gy). For all colony formation assays, cells were exposed to the drug for the duration of the experiment. All experiments were conducted three independent times, with the mean ± SEM reported. *p<0.05; ***p<0.001; two-way ANOVA. Combination of Dacomitinib plus RT demonstrated a synergistic interaction across all dosing regimens in all three SCCHN cell lines (Chou-Talalay Method).

Colony formation assays were similar to the MTS data in that the FaDu cells were the least sensitive to Dacomitinib, followed by UT-SCC-8 and UT-SCC-42a cells (Figure 3A). A dose-dependent reduction in clonogenic survival was observed in all SCCHN lines, with a significant reduction in survival being observed with as low as 10 nM of Dacomitinib. This effect was significantly enhanced when Dacomitinib (5–100 nM) was combined with IR (2 or 4 Gy). Analysis of the normalized isobolograms prepared using the Chou-Talalay Synergy Quantification Method [29] revealed that the combination of Dacomitinib with IR resulted in a synergistic interaction for all three SCCHN cell lines (Figure S1 in File S1).

### Dacomitinib inhibited EGFR signalling and phosphorylation of downstream targets in vitro

**Figure 4:**
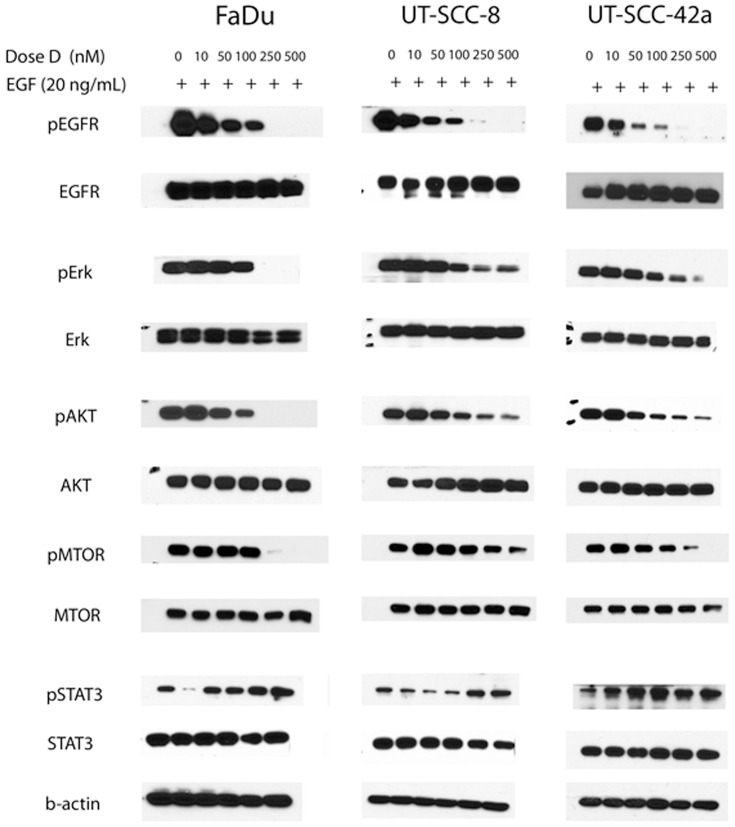
Treatment of SCCHN cells with Dacomitinib inhibited EGFR signalling. FaDu, UT-SCC-8, and UT-SCC-42a cells were treated with Dacomitinib (D; 0, 10, 50, 100, 250, or 500 nM) in serum-free media for 24 hours. Thirty minutes prior to lysis, cells were stimulated with EGF (20 ng/mL). Experiments were performed three independent times, with similar results; representative blots are shown.

To verify that the observed cytotoxicity induced by Dacomitinib was mediated by EGFR inhibition, Western blot analysis was conducted for the three SCCHN cell lines. All three SCCHN cell lines expressed basal EGFR, as well as the downstream AKT, ERK, mTOR, and STAT3, both with and without exogenous EGF (Figure 4, Figure S2 in File S1). Treatment with Dacomitinib resulted in a dose-dependent reduction of p-EGFR in all three SCCHN cell lines, with or without EGF stimulation (Figure 4, Figure S2 in File S1). Focusing on the samples stimulated with EGF, >90% inhibition of EGFR phosphorylation was observed with 250 nM of Dacomitinib for all three SCCHN cell lines (Figure 4). Based on densitometry analysis, the IC50 (concentration of Dacomitinib that reduces phosphorylation by 50%, as compared to the untreated control) values for EGFR phosphorylation were 100, 50, and 100 nM for FaDu, UT-SCC-8, and UT-SCC-42a cell lines, respectively (Figure S3 in File S1). The IC50 concentrations of Dacomitinib for downstream phosphorylation of ERK, AKT, and mTOR were all in the range of 50 to 250 nM for all three cell lines. Interestingly, STAT3 phosphorylation was significantly reduced at lower concentrations of Dacomitinib (10 nM), but was induced to greater than untreated levels when higher concentrations of Dacomitinib were utilized for all three cell lines (Figure 4).

### Dacomitinib induced G1 cell cycle arrest in SCCHN cells in vitro

**Figure 5:**
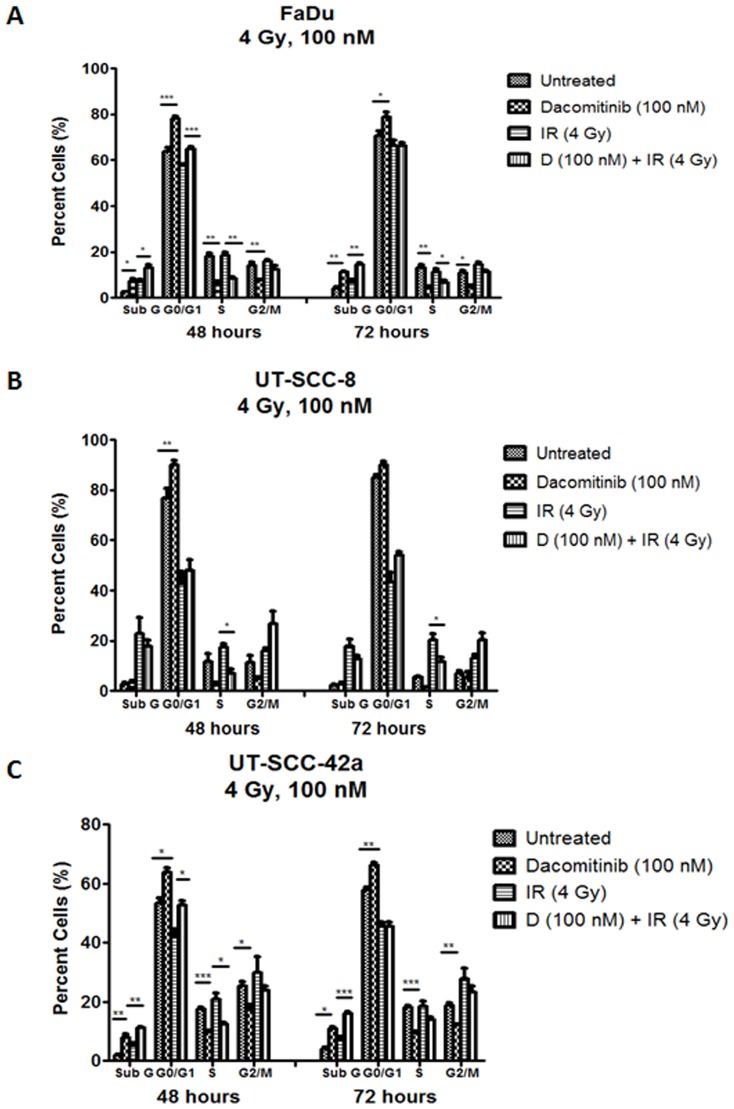
Dacomitinib, with or without ionizing radiation, perturbed the cell cycle in SCCHN cells. (A-C) FaDu, UT-SCC-8, and UT-SCC-42a cells were treated with negative control (DMSO), Dacomitinib (100 nM), IR (4 Gy), or Dacomitinib plus IR (100 nM; 4 Gy). Cells were fixed at 48 or 72 hours post-treatment, then analysed. The plots represent data from three independent experiments, with the mean ± SEM reported. *p<0.05; **p<0.01; ***p<0.001; Student's *t* test.

To elucidate the mode of cytotoxicity of Dacomitinib, cell cycle analyses were performed on SCCHN cell lines treated with Dacomitinib (10 or 100 nM), either alone or in combination with IR (2 or 4 Gy). Forty-eight hours following Dacomitinib treatment, there was a dose-dependent increase in the population of cells in G0/G1, along with a corresponding reduction in the proportion of cells in the S and G2/M phases, again observed across all three SCCHN cell lines (Figure S4 in File S1). Furthermore, an increase in the sub-G1 population in both the FaDu (7.5% vs. 2.6% for untreated) and UT-SCC-42a (8.6% vs. 1.9% for untreated) cells was observed at the 100 nM Dacomitinib concentration, compared to untreated counterparts. Combining Dacomitinib (100 nM) with IR (4 Gy) resulted in an increase in the proportion of G0/G1 cells in all three SCCHN cell lines, as compared to IR alone (Figure 5). Irradiation further enhanced the percentage of sub-G1 cells in Dacomitinib-treated FaDu and UT-SCC-42a cells.

To further elucidate the mechanism of Dacomitinib-induced cytotoxicity *in vitro*, DNA damage and senescence were also assessed (Supporting Materials and Methods in File S1). DNA double strand breaks were analyzed using, phosphoryation of γ-H2AX (p-γ-H2AX) as the read-out [25]; no significant difference in p-γ-H2AX levels was observed in cells treated with Dacomitinib plus IR, compared to IR alone (data not shown). In order to detect possible Dacomitinib-induced SCCHN senescence, senescence-associated β-galactosidase was assessed using various concentrations of Dacomitinib (0–500 nM) (Supporting Materials and Methods in File S1); again, senescence was not observed for up to 7 days post-treatment in all three SCCHN cell lines (data not shown).

### Combining Dacomitinib and ionizing radiation enhanced tumor growth delay in vivo

**Figure 6:**
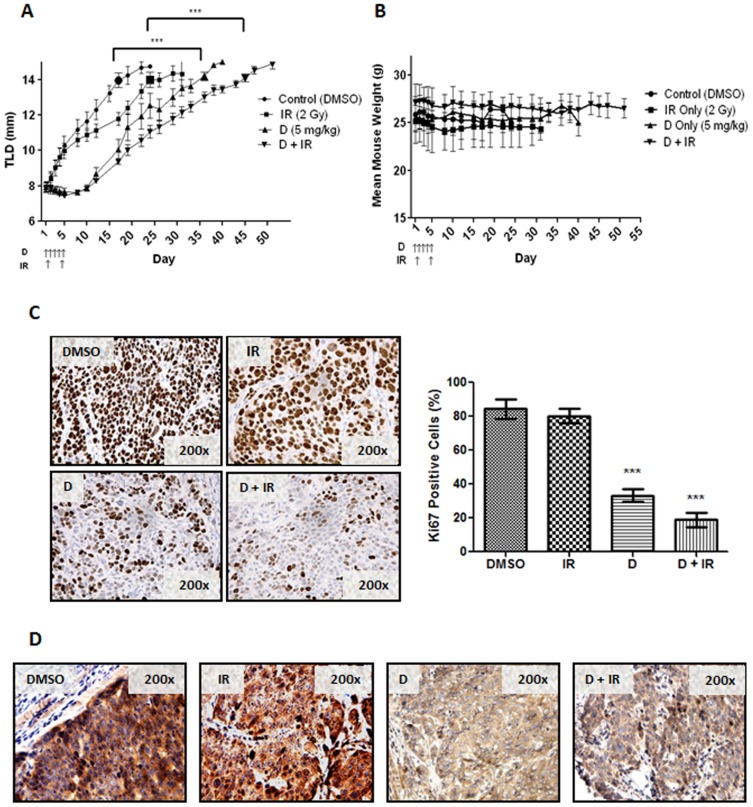
Dacomitinib delayed FaDu tumor growth, alone and in combination with IR. (A) FaDu-bearing mice were randomized to: (i) DMSO control; (ii) Dacomitinib (5 mg/kg/d) delivered orally on days 1 to 5; (iii) local tumor irradiation (IR; 2 Gy/treatment) delivered on days 2 and 5; or (iv) Dacomitinib plus IR. Mean tumor leg diameter (TLD) from three independent experiments (three mice per treatment group, per experiment) is reported ± SEM. ***p<0.001, negative control *vs*. Dacomitinib only, or RT only *vs*. Dacomitinib plus RT; Student's *t* test. (B) Mean mouse weight ± SEM from experiment (A). (C) Ki-67 staining was performed on treated FaDu tumors: DMSO, radiation (IR), Dacomitinib (D), or Dacomitinib plus radiation (D+IR), as from (A). Tumors were extracted from mice 24 hours after the final treatment. Ki-67 positive cells stain dark brown. The right-hand panel represents the proportion of Ki-67-positive cells for each treatment group. Ki-67 scoring was conducted by counting the number of positive tumor cells in 3 representative sections for each tumor. Mean ± SEM is reported. ***p<0.001; Student's *t* test. (D) p-EGFR staining was performed on paraffin sections, as in (C). Positive Staining for p-EGFR is brown.

To assess the pre-clinical efficacy of Dacomitinib in combination with IR *in vivo*, FaDu cells were used to generate xenograft tumors in SCID mice that were subsequently randomized into four treatment groups: vehicle control, Dacomitinib alone, IR alone, and Dacomitinib plus concurrent IR treatment. As shown in Figure 6A, vehicle control mice were sacrificed between 17–22 days post-treatment. Dacomitinib treatment alone significantly improved the mean time until endpoint (TLD of 14 mm) by 13 days, as compared to the control group (p<0.001; Figure S5 in File S1). The greatest tumor growth delay was observed for the IR with concurrent Dacomitinib-treated mice, whereby the combined treatment regimen improved the mean time until endpoint by 26 days, as compared to control mice (p<0.001; Figure S5 in File S1). All four treatment regimens were well tolerated, as total mouse body weights remained unchanged (<15% fluctuation) for the 50-day duration of these experiments (Figure 6B).

### Dacomitinib reduced tumor cell proliferation and EGFR activation in vivo

Immunohistochemical analyses of the xenograft tumors were performed to determine if the observed *in vivo* effects were mediated by EGFR inhibition. Tumors were removed 24 hours after the last treatment (day 6), then stained for TUNEL, Ki-67, and p-EGFR. Radiation alone had minimal effects on Ki-67 immunostaining (80% positive cells for IR alone *vs*. 83% positive cells for negative control); however, a significant reduction was observed for Dacomitinib alone (33% positive cells; p<0.001; Figure 6C), which was further enhanced with the addition of IR (19% positive cells; p<0.001; Figure 6C.). Similarly, p-EGFR was intensely expressed in the vehicle control and IR only tumors, which was significantly reduced when tumors were treated with Dacomitinib alone, as well as Dacomitinib plus IR (Figure 6D). There was no significant difference in TUNEL-positivity (Vehicle Control 6.2±2.0%; Dacomitinib only 7.7±0.6%; IR only 10.2±2.0%; Dacomitinib + IR 9.5±0.4%)

## Discussion

This is the first report evaluating the potential role of Dacomitinib for the treatment of SCCHN in combination with IR. Dacomitinib was highly effective at blocking EGFR phosphorylation in all three SCCHN cell lines, resulting in decreased proliferation, reduced clonogenicity, along with a significant increase in G1 cell cycle arrest *in vitro*. These findings were corroborated *in vivo*, as Dacomitinib reduced both EGFR phosphorylation and tumor cell proliferation, resulting in delayed tumor growth. Furthermore, the interaction between Dacomitinib and IR was synergistic *in vitro*, and additive *in vivo*.

Dysregulation of the EGFR pathway has been well documented in many human malignancies. In SCCHN, nearly all tumors harbour EGFR over-expression, the extent of which relate to resistance to chemotherapy and/or radiation, leading to decreased survival [10], [11], [30]. Over-expression of the other ErbB receptors in SCCHN has been reported (ErbB2, 3–29%; ErbB3, 21%; ErbB4, 26%), and can also contribute to disease progression [4]. In the three SCCHN cell lines evaluated in this current study, EGFR was uniformly over-expressed, but basal expression of the other three members of the ErbB family was not observed to be increased (Figure 1A).

Inhibition of EGFR has been examined extensively for the treatment of SCCHN, with previous reports concluding that EGFR is an attractive therapeutic target [12], [13], providing the rationale for the Cetuximab plus RT trial [14]. However, clinical response to Cetuximab plus RT, although significant, has remained modest [31], [32], motivating the search for improved therapeutic strategies, such as a pan-ErbB inhibitor. Indeed, this study evaluating Dacomitinib demonstrated significant dose-dependent inhibition of EGFR phosphorylation (Figure 4) and its well-described downstream mediators: the RAS/RAF/MEK/ERK and PI3K/AKT/mTOR pathways [33]. Interestingly, activation of STAT3, which plays a key role in regulating cell growth and apoptosis, was significantly reduced at a low dose of Dacomitinib (10 nM), but appeared to be induced with higher doses (Figure 4). EGFR is obviously a known upstream activator of STAT3, through phosphorylation of the SRC tyrosine kinase [34]. However, EGFR-independent STAT3 signalling also exists, through activation of the glycoprotein 130 (gp130) receptor family *via* interleukin 6 (IL-6), described for SCCHN cell lines [35]. The combination of EGFR with STAT3 inhibitors has shown improved anti-tumor activity in other pre-clinical models [36]; certainly, data from the current study would support further investigation of combining Dacomitinib with a STAT3 inhibitor for SCCHN. Indeed, the combination of EGFR with SRC inhibitors has already been suggested to be of potential benefit for patients with SCCHN [12].

Given that RT is a primary curative modality for SCCHN, the effects of Dacomitinib with IR were examined; consistently demonstrating a synergistic interaction *in vitro* (Figure 2 and Figure 3), and an additive effect *in vivo* (Figure 6). Ionizing radiation alone can induce EGFR activation, through increased expression of either EGFR or its ligand TGF-α, which may then lead to a proliferative response [37]. Other EGFR inhibitors have been combined with IR, all consistently supporting this combinatorial strategy for anti-tumor efficacy [38]–[40]. Multiple mechanisms of radio-sensitization by EGFR inhibition have been described, including cell cycle perturbation [41], inhibition of DNA damage repair [42], as well as an anti-angiogenic effects [43]. Our current study demonstrated the expected dose-dependent increase in G1 cell cycle arrest after treatment with Dacomitinib (Figure S4 in File S1), which was consistent with the findings of Ather *et al* [17]. A corresponding decrease in the proportion of cells in S phase, a more radio-resistant phase [44], was also observed. This was corroborated *in vivo* by the significant reduction in Ki-67 immuno-expression (Figure 6C). In two of the three cell lines (FaDu and UT-SCC-42a), the combination of Dacomitinib with IR demonstrated an increase in the sub-G1 fraction, suggesting enhanced apoptosis (Figure 5), although this was not corroborated in the xenograft model. These results are concordant with previous reports evaluating the effects of EGFR inhibitors with IR in other SCCHN cancer models [45], [46], supporting the applicability of Dacomitinib in combined treatment regimens that include IR.

One of the persistent challenges to targeting EGFR has been the intrinsic or acquired resistance to current EGFR inhibitors, linked to a variety of mechanisms. For example, increased ErbB2 and ErbB3 activation can arise from Cetuximab therapy, which may be responsible for acquired resistance [47], [48]. These studies identified resistance mechanisms linked to bypass signalling, wherein the ErbB pathway remains activated despite EGFR inhibition. Hence, the ability of Dacomitinib to effectively inhibit the kinase activity of not only EGFR, but also ErbB2 and ErbB4, should theoretically abrogate this bypass mechanism of resistance [17], [18]. This was already demonstrated wherein Cetuximab-resistant SCCHN cell lines were re-sensitized to EGFR inhibition once ErbB2 and/or ErbB3 inhibitors were added to Cetuximab [47].

Activating mutations of EGFR have been shown to correlate with response to EGFR inhibition in other human malignancies. While some mutations can confer sensitivity to EGFR targeting, such as the G719C and the L858R mutations, others like the T790M mutation have, in fact, been correlated with resistance [49], [50]. These mutations, however, are rare in SCCHN [51], and were not found to be present in the three SCCHN cell lines used in this study (Table S2 in File S1). Perhaps a more clinically relevant mutation for SCCHN might be the EGFR variant (vIII) mutation, wherein the extracellular ligand binding domain of EGFR contains an in-frame deletion of exons 2–7, rendering EGFR constitutively activated [52]. The EGFR vIII mutation has been observed in approximately 40% of SCCHN patients, associated with chemotherapy resistance and decreased growth inhibition in response to Cetuximab [52]–[54]. All three SCCHN cell lines used in this study were negative for the EFGR vIII mutation (data not shown); although further investigation to assess the efficacy of Dacomitinib in such SCCHN models harbouring the EGFR vIII mutation would certainly be warranted.

There has been a steady emergence of data describing HPV associated SCCHN as a distinct sub-type of this disease. Clinically, HPV-positive SCCHN predominately presents in the oropharynx, and HPV-positive patients demonstrate better response rates to treatment and improved overall and disease free survival [55]. In this study, our cell lines were derived from hypopharyngeal (FaDu) and laryngeal (UT-SCC-8 and UT-SCC-42a) SCC's and are all HPV-negative. Further investigation into the effects of Dacomitinib treatment, with or without IR, in an HPV-positive SCCHN model should be undertaken. However, recent evidence suggests that HPV oncogenes may not have an effect on the EGFR pathway in SCCHN and that the response to anti-EGFR treatment may be independent of HPV status in patients with SCCHN [56].

In conclusion, this study demonstrated that Dacomitinib is an effective anti-proliferative agent for the treatment of SCCHN, both *in vitro* and *in vivo*. The inhibition of EGFR phosphorylation by Dacomitinib resulted in reduced downstream signalling of AKT, ERK, and mTOR, leading to decreased cell viability and clonogenic survival, and an increase in G1 cell cycle arrest *in vitro*. The combination of Dacomitinib with IR, was synergistically cytotoxic *in vitro*. The anti-proliferative effects of Dacomitinib alone were confirmed *in vivo*, as well as its additive anti-tumor efficacy when combined with IR, with no apparent additional toxicity on normal tissues. Hence, these data strongly support the further evaluation of Dacomitinib combined with radiation in early phase studies for patients with SCCHN.

## Supporting Information

File S1 Contains: Supporting Materials and Methods. Table S1. Table S2. Figure S1. Figure S2. Figure S3. Figure S4. Figure S5.
(DOCX)
