# Supplementary material for: Pre-Clinical Characterization of Dacomitinib (PF-00299804), an Irreversible Pan-ErbB Inhibitor, Combined with Ionizing Radiation for Head and Neck Squamous Cell Carcinoma
Source: PLoS One. 2014 May 22;9(5):e98557. doi: 10.1371/journal.pone.0098557 (PMC4031184; doi:10.1371/journal.pone.0098557)
Supplement: File S1 — Contains: Supporting Materials and Methods. Table S1. Table S2. Figure S1. Figure S2. Figure S3. Figure S4. Figure S5. (DOCX) [file pone.0098557.s001.docx]

**SUPPORTING MATERIALS AND METHODS**

***Quantification of Phosphorylated λ-H2AX***

Cells were seeded in 6-well plates at a density of 3 x 10^5^ cells per well. 24 hours post-seeding, cells were treated with negative control (0.01% DMSO) or Dacomitinib (10 or 100 nM). One hour post-treatment, cells were exposed to 4 Gy. Cells were then harvested at various time points after radiation treatment and stained for phosphorylated histone H2AX (y-H2AX), as previously described. Briefly, cells were suspended in 500 µL FITC-labelled mouse monoclonal anti-phosphorylated y-H2AX antibody (1:500 dilution in TBS-T; Upstate Biotechnology) for two hours at room temperature, washed with TBS, and then analyzed using the FACSCalibur (BD).

***EGFR Mutation Status***

In order to assess the mutational status of the three SCCHN cell lines, as well as the NOE cell line, four commonly mutated regions within the EGFR gene were chosen for sequencing. Briefly, cells were seeded in 6 well plates at a density of 5 x 10^5^ in media. Forty-eight hours post-seeding, genomic DNA was isolated from cells using the DNeasy Blood and Tissue Kit (Qiagen). Specific primers for the regions of interest were designed using Primer3 (NCBI; Table S2) and PCR was performed using Platinum Taq polymerase (Invitrogen), according to manufacturer’s specifications. PCR products were run on a 1.0% agarose gel and extracted for sequencing using the PureLink quick gel extraction kit (Invitrogen). DNA was sent to the Sequencing Facility at the Sick Kid’s Hospital, Toronto, ON (The Centre for Applied Genomics; TCAG) and sequence results were compared to the respective known gene sequences (NCBI).

***Senescence-Associated β-Galactosidase***

Cells were seeded in 12 well plates at a density of 5 x 10^4^ cells per well. Twenty-four hours post-seeding, cells were treated with negative control (0.01% DMSO) or Dacomitinib (10, 25, 50, 100, 250, or 500 nM), followed by ionizing radiation (0, 2, 4 Gy) within one hour of drug treatment. After incubation for 48, 72, or 168 hours, media containing Dacomitinib or DMSO was removed from each well and cells were washed with PBS. Cells were fixed and stained using the Senescence β-Galactosidase Staining Kit (#9860, Cell Signalling), according to the manufacturer’s specifications. The percentage of positively stained cells was calculated from 5 separate and representative microscopic fields (200x) and compared to the untreated control.

**SUPPORTING TABLES AND FIGURES**

**Table S1. qRT-PCR primers used for ErbB mRNA expression levels.** Gene specific primers were designed to determine mRNA transcript levels for EGFR, ErbB2, ErbB3, and ErbB4 in SCCHN and NOE cell lines. Primers were designed using Primer3 (NCBI).

| **Target Gene** | **Forward Primer** | **Reverse Primer** |
| --- | --- | --- |
| EGFR | 5’-GAC TGC CTG GTC TGC CGC AA-3’ | 5’-GGA CGC ACG AGC CGT GAT CT-3’ |
| ErbB2 | 5’-GAC ACC AAC CGC TCT CGG GC-3’ | 5’-ACA GAC AGT GCG CGT CAG GC-3’ |
| ErbB3 | 5’-CCT GAG TGT GAC CGG CGA TGC-3’ | 5’-TCG GCA TTG TGT CCC GTG AGC-3’ |
| ErbB4 | 5’-ACA GCA GTA CCG AGC CTT GCG-3’ | 5’-ACC GCA GGA AGG AGA GGT CCC-3’ |
| β-actin | 5’-CCC AGA TCA TGT TTG AGA CCT-3’ | 5’-AGT CCA TCA CGA TGC CAG T-3’ |

**Table S2. EGFR mutational status in human head and neck cell lines.** Selected regions of the EGFR gene were sequenced from PCR products obtained using genomic DNA isolated from the normal oral epithelial (NOE) cell line, as well as the three SCCHN cell lines FaDu, UT-SCC-8, and UT-SCC-42a. Specific primers were designed to flank the region(s) of interest. PCR products were run on an agarose gel and extracted for sequencing.

| **Exon** | **Mutation Site(s)** | **Primer Sequences** |  | **Mutation Status** | |  |
| --- | --- | --- | --- | --- | --- | --- |
|  |  |  | **NOE** | **FaDu** | **UT-SCC-8** | **UT-SCC-42a** |
| 18 | G719C | F- 5’ CAA ATG AGC TGG CAA GTG CCG TGT C 3’  R- 5’ GAG TTT CCC AAA CAC TCA GTG AAA C 3’ | w/t | w/t | w/t | w/t |
| 19 | K745 -I759 (del) | F- 5’ GCA ATA TCA GCC TTA GGT GCG GCT 3’  R- 5’ CAT AGA AAG TGA ACA TTT AGG ATG TG 3’ | w/t | w/t | w/t | w/t |
| 20 | T790M | F- 5’ CCA TGA GTA CGT ATT TTG AAA CTC 3’  R- 5’ CAT ATC CCC ATG GCA AAC TCT TGC 3’ | w/t | w/t | w/t | w/t |
| 21 | L858R, L861Q | F- 5’ CTA ACG TTC GCC AGC CAT AAG TCC 3’  R- 5’ GCT GCG AGC TCA CCC AGA ATG TCT GG 3’ | w/t, w/t | w/t, w/t | w/t, w/t | w/t, w/t |

**
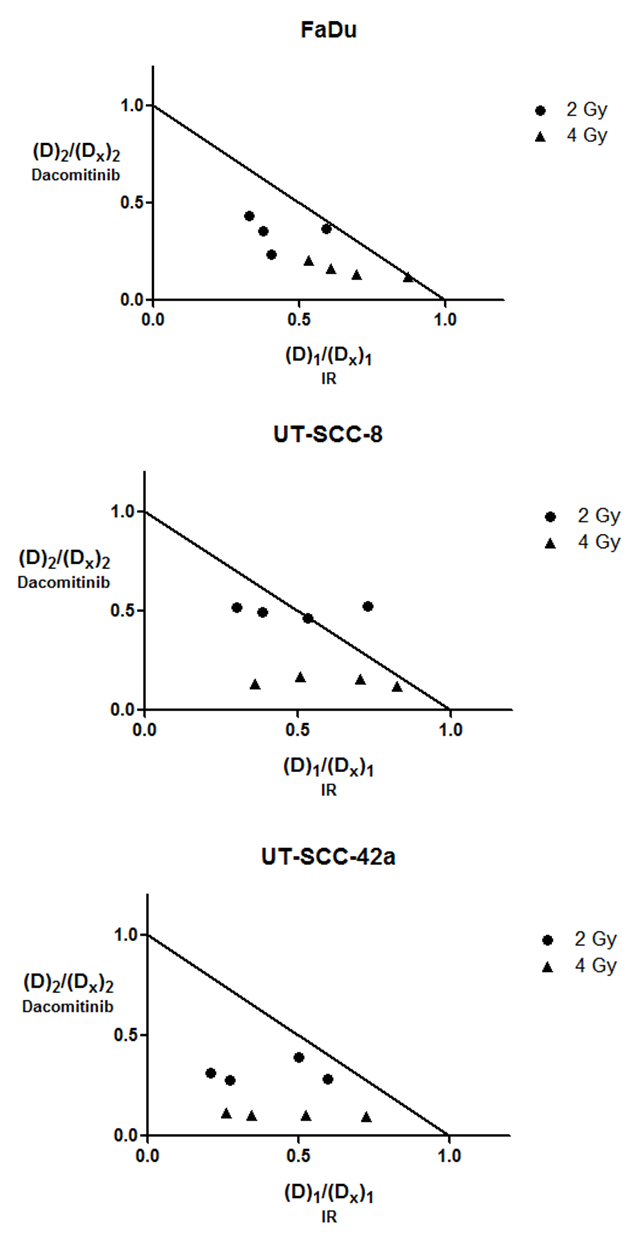
**

**Figure S1. Dacomitinib treatment combined with ionizing radiation inhibits clonogenic survival in a synergistic manner.** (A-C) Normalized isobolograms for non-constant combination ratios prepared using the Chou-Talalay synergy quantification method. Clonogenic survival data obtained from colony formation assays (Figure 2) was analysed to assess the interaction between Dacomitinib and ionizing radiation, when used in a bi-modality treatment regimen, in FaDu, UT-SCC-8, and UT-SCC-42a SCCHN cell lines. Plotted line represents perfectly additive interaction; points falling to the left of the plotted line indicate synergistic interaction, whereas points falling to the right of the plotted line indicate an antagonistic interaction.


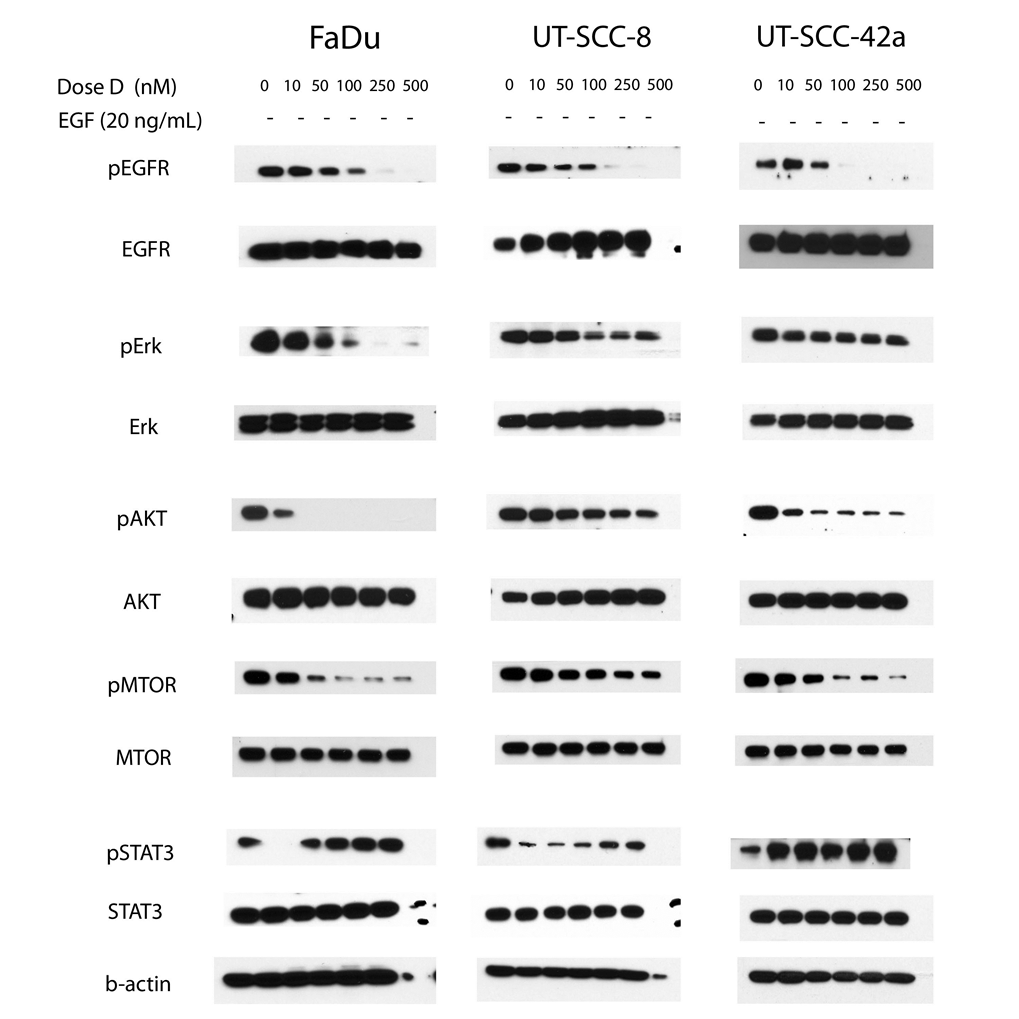


**Figure S2. Dacomitinib treatment reduces EGFR signalling in SCCHN cell lines.** FaDu, UT-SCC-8, and UT-SCC-42a cells were treated with increasing concentrations of Dacomitinib (D; 0-500 nM) in serum free media for twenty-four hours. Cells were lysed without receiving EGF stimulation to assess the basal level of signalling. Experiments were performed independently three times, with similar results. Representative blots shown.

**
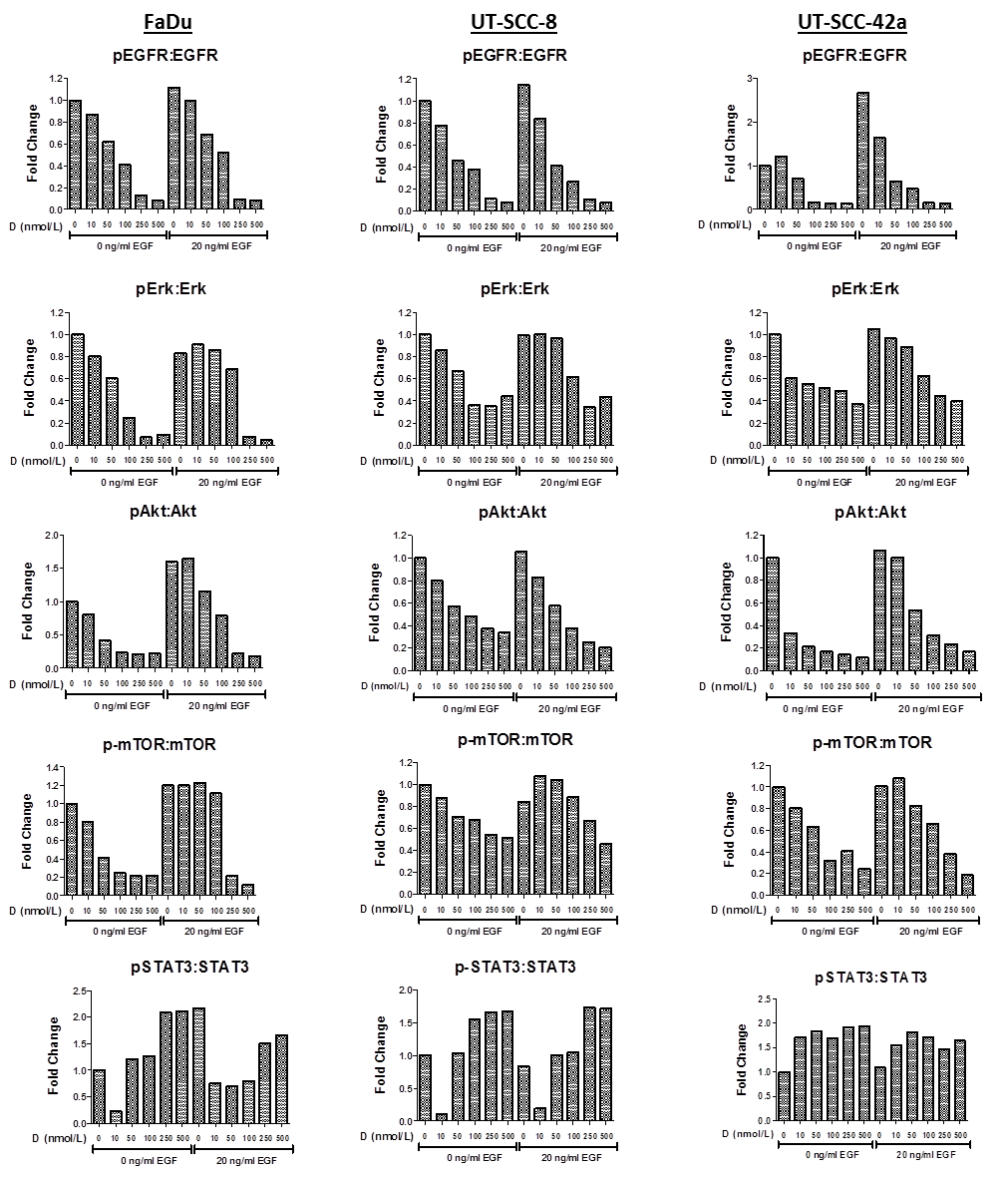
**

**Figure S3. Dacomitinib treatment reduces EGFR signalling in SCCHN cell lines, both with and without exogenous EGF stimulation.** Densitometry analysis of Western blots. Signal intensities (obtained using Adobe Photoshop) were first normalized to β-actin for each cell line. The ratio of phosporylated signal to total signal was calculated, and the basal signalling ratio (no EGF, no drug) was set to 1 to determine signal fold change.

**
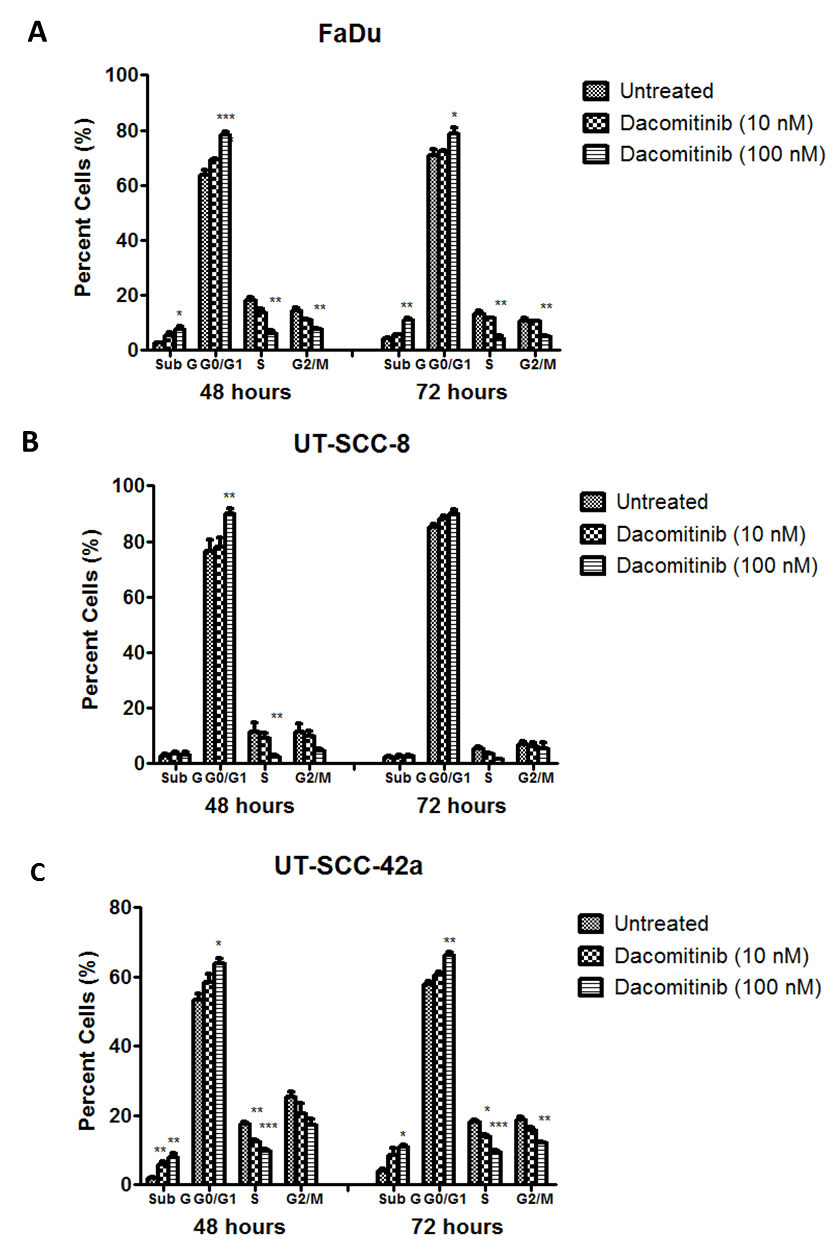
**

**Figure S4. Cell cycle profiles of 3 SCCHN cell lines treated with Dacomitinib.** (A-C) FaDu, UT-SCC-8, and UT-SCC-42a were treated with either negative control, 10 nM, or 100 nM Dacomitinib. Cells were fixed at forty-eight and seventy-two hours post treatment, stained with propidium iodide, and analysed using BD FACSCalibur. Data was analysed using FlowJo software (Tree Star). Graphs represent data from three independent experiments, with the mean ± SEM reported. * p<0.05, ** p<0.01, *** p<0.001.

**
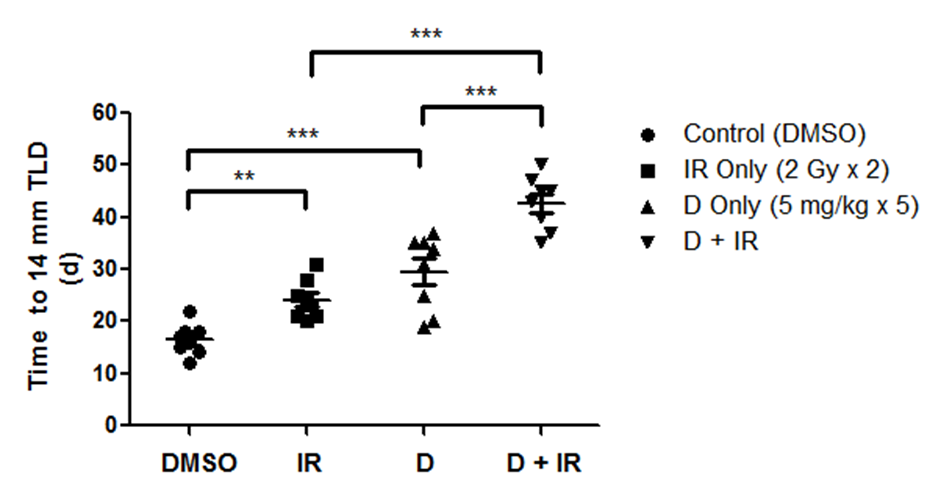
**

**Figure S5. Combining Dacomitinib with ionizing radiation results in greatest tumor growth delay, *in vivo*.** The FaDu xenograft model was used to perform the tumor growth delay assay (Fig 5). Mean time until endpoint (14 mm, tumor plus leg diameter (TLD)) was calculated for the following treatment groups: a) negative control (DMSO), b) ionizing radiation only (IR), c) Dacomitinib only (D), and d) D + IR. Data points represent number of days until endpoint for each mouse in treatment group. Error bars indicate ± SD. ** p<0.01, *** p<0.001; Student’s t test.
